# Laminography as a tool for imaging large-size samples with high resolution

**DOI:** 10.1107/S1600577524002923

**Published:** 2024-05-21

**Authors:** Viktor Nikitin, Gregg Wildenberg, Alberto Mittone, Pavel Shevchenko, Alex Deriy, Francesco De Carlo

**Affiliations:** a Advanced Photon Source Argonne National Laboratory Lemont IL60439 USA; b University of Chicago Chicago IL60637 USA; Paul Scherrer Institut, Switzerland

**Keywords:** laminography, reconstruction, GPU, neuroimaging

## Abstract

Despite the increased brilliance of the new generation synchrotron sources, there is still a challenge with high-resolution scanning of very thick and absorbing samples, such as a whole mouse brain stained with heavy elements, and, extending further, brains of primates. Samples are typically cut into smaller parts, to ensure a sufficient X-ray transmission, and scanned separately. Compared with the standard tomography setup where the sample would be cut into many pillars, the laminographic geometry operates with slab-shaped sections significantly reducing the number of sample parts to be prepared, the cutting damage and data stitching problems. In this work, a laminography pipeline for imaging large samples (>1 cm) at micrometre resolution is presented. The implementation includes a low-cost instrument setup installed at the 2-BM micro-CT beamline of the Advanced Photon Source. Additionally, sample mounting, scanning techniques, data stitching procedures, a fast reconstruction algorithm with low computational complexity, and accelerated reconstruction on multi-GPU systems for processing large-scale datasets are presented. The applicability of the whole laminography pipeline was demonstrated by imaging four sequential slabs throughout an entire mouse brain sample stained with osmium, in total generating approximately 12 TB of raw data for reconstruction.

## Abstract 2

A laminography setup and software toolbox for efficient imaging of large-size samples are presented. Applicability of the developed technique is demonstrated by imaging slabs through a whole mouse brain sample stained with heavy elements.

## 1. Introduction

The computed laminography technique is an extension of regular computed tomography, which involves tilting the rotary stage along the beam direction (Hasenkamp, 1973 ▸; Gondrom *et al.*, 1999 ▸). This allows for scanning planar and high X-ray absorbing samples with enhanced quality and less radiation damage.

Laminography imaging has been demonstrated at several synchrotron facilities and lab-CT systems around the world. Helfen *et al.* implemented the technique at several beamlines of the European Synchrotron Radiation Facility (ESRF) (Helfen *et al.*, 2005 ▸, 2011 ▸, 2013 ▸). The method has become a routine tool at the synchrotron by showing high-quality results for many kinds of samples (Xu *et al.*, 2010 ▸; Reischig *et al.*, 2013 ▸; Morgeneyer *et al.*, 2014 ▸). Recently, the first *in situ* nano-laminography has been demonstrated using the Projection X-ray Microscope at the ESRF (Hurst *et al.*, 2023 ▸). Furthermore, multi-contrast computed laminography was shown at a beamline of Karlsruhe Research Accelerator (Cheng *et al.*, 2013 ▸) – the authors used a grating interferometer to enhance phase contrast of a butterfly sample. Hoshino *et al.* demonstrated laminography at SPring-8 by analysing a copper grid pattern with alphabetical letters (Hoshino *et al.*, 2011 ▸). At the Swiss Light Source, the laminography geometry has been also used in nano-resolution 3D ptychographic imaging of integrated circuits (Holler *et al.*, 2019 ▸, 2020 ▸). Laminography has become popular also for lab-CT sources with cone X-ray beams. Different cone-beam laminography trajectories were compared by O’Brien *et al.* (2016 ▸). Fisher *et al.* (2019 ▸) demonstrated a computed laminography implementation on a conventional industrial laboratory micro-CT scanner (Nikon XTEK), without the need for special equipment. The authors also presented methods for reducing laminography artefacts due to insufficient sampling. Another custom build X-ray compued tomography (CT) scanner was introduced by Deyhle *et al.* (2020 ▸), together with a detailed guidance on the instrument calibration and optimal data acquisition. Furthermore, recent progress in robotic sample manipulator systems has facilitated the adjustment of laminography scanning geometry (Wood *et al.*, 2019 ▸).

Several reconstruction software packages have functionality for laminography reconstruction. In the *Astra Tomography Toolbox* (van Aarle *et al.*, 2015 ▸), the laminography geometry can be explicitly defined, followed by running iterative reconstruction (*e.g.* SIRT or CGLS method) on GPUs. For an iterative method, necessary data are typically kept in GPU memory during all iterations, minimizing the overhead for data copy between the CPU and GPU memory. In such cases, the performance of the reconstruction is mostly limited by the GPU computation speed. Another package, called *UFO* (Faragó *et al.*, 2022 ▸), provides a multi-threaded, GPU-enabled and distributed data processing framework for tomographic and laminographic reconstruction. Both packages, *Astra* and *UFO*, implement laminography reconstruction by direct discretization of the forward and backward projection line integrals. In this case, computational complexity is 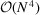 assuming that the number of projection angles and volume size in each dimension are of the order of *N*.

Computational complexity for laminography reconstruction can be decreased to 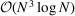 using a Fourier-based method and fast Fourier transform (FFT), similar to the one used for regular tomography with the Gridrec algorithm (Dowd *et al.*, 1999 ▸) in *TomoPy* (Gürsoy *et al.*, 2014 ▸), or by *Fourierrec* in *TomocuPy* (Nikitin, 2023 ▸). The tomography back-projection operator can be rapidly evaluated as a combination of the one-dimensional FFT and two-dimensional unequally spaced inverse FFT (Beylkin, 1998 ▸). In turn, rapid evaluation of the laminography back-projection operator can be performed as a combination of the two-dimensional FFT and three-dimensional unequally spaced inverse FFT (Myagotin *et al.*, 2013 ▸; Voropaev *et al.*, 2016 ▸). Computational complexity plays an important role when reconstructing data obtained by stitching several projection datasets (Vescovi *et al.*, 2018 ▸). For instance, performance tables in Nikitin (2023 ▸) show that tomographic reconstruction by a method with lower complexity for 2048^3^, 4096^3^ and 8192^3^ volumes becomes faster than the direct discretization by factors of 5, 10 and 20, respectively. Current data storage allows working with stitched data of more than 16384 pixels in each dimension (more than 16 TB in single precision); therefore algorithms with lower computational complexity indeed become critical for any future tomography and laminography applications.

Nvidia GPUs have demostrated to be an essential tool in accelerating computational imaging programs. VRAM (current maximum is 80 GB for Tesla H100) is significantly smaller than computer RAM, therefore in most cases additional data splitting and transfer mechanisms have to be performed before reconstruction. These mechanisms are straightforward for regular computed tomography since each *z*-slice can be processed independently. But in laminography, more complex data handling procedures have to be developed since there is a dependence between slices due to the tilted geometry. Moreover, laminography slabs typically have larger sizes in two dimensions, that do not fit the detector field of view. This requires the implementation of a mosaic scanning protocol, where the slab is scanned at different positions and projection data are stitched to form a large data volume for further reconstruction. This is why it is fundamental to have fast GPU-based reconstruction with low computational complexity.

In this work, we consider the laminography technique as a tool for optimal scanning of large-size samples. Imaging very thick and absorbing samples requires cutting these samples into parts to ensure sufficient X-ray transmission, and scanning each part independently. Compared with the standard tomography setup where the sample would be cut into pillars, the laminographic geometry uses slab-shaped samples, which requires a significantly lower number of cutting procedures. With proper slab thickness, the X-ray propagation distance through the slab can be sufficient to obtain acceptable photon counts for different materials.

Our contribution through this paper can be summarized as follows:

(i) Description of the synchrotron laminography setup. We report how laminography is implemented at the micro-CT beamline 2-BM of the Advanced Photon Source (APS), USA. The simplicity and adaptability of this setup make it an ideal choice for implementation at other synchrotron beamlines.

(ii) GPU-based laminography reconstruction. We implement laminography reconstruction using the Fourier-based method [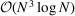] on GPU with efficient asynchronous data processing by chunks where CPU–GPU data transfers and GPU processing are timely overlapped almost fully hiding time for data transfers.

(iii) Integrating laminography with *TomocuPy.* We add the optimized laminography reconstruction in the *TomocuPy* package (https://tomocupy.readthedocs.io – in the ‘develop’ github branch during the paper review). *TomocuPy* provides an easy-to-use command-line interface for GPU-based reconstruction. Besides reconstruction of full volumes, it also provides functionality for adjusting the rotation axis and laminography tilt angle.

(iv) Iterative reconstruction with regularization. Fast implementation of forward and adjoint laminography operators can be used for constructing iterative schemes with regularization. We provide, as an independent package, the implementation of reconstruction with total variation regularization for suppressing laminography artefacts due to insufficient sampling.

(v) Scanning and reconstruction of large samples with laminography. Laminography simplifies the sample preparation process by requiring significantly less cutting compared with regular tomography, although more complex data stitching and reconstruction techniques are required in the case of large samples. We will describe the whole pipeline employed with large samples using as an example the imaging of four sequential mouse brain sections with micrometre resolution. The brain section datasets are made available in TomoBank (De Carlo *et al.*, 2018 ▸) (see https://tomobank.readthedocs.io/ under Laminography/Brain).

## 2. Strategy for scanning large-size samples

We will start with a brief discussion on strategies for scanning large-size samples with high resolution. As an example let us consider micro-resolution imaging of an adult mouse brain sample. A typical mouse brain has a size of 12 mm (anterior to posterior) × 11 mm × 8 mm (dorsal to ventral). To increase X-ray absorption contrast in the projections, the brain is often stained with heavy metals (osmium, lead) (Hua *et al.*, 2015 ▸) especially if further investigations (*i.e.* electron microscopy) are required. Nevertheless, a high concentration of heavy materials leads to strong X-ray beam absorption by the sample. In this case, a possible solution is to use a higher energy X-ray beam, which however also significantly affects the achievable spatial resolution and sensitivity to variations in attenuation coefficient for large samples (Grodzins, 1983 ▸; Flannery *et al.*, 1987 ▸). Based on our calculation, scanning the stained mouse brain may require energy of more than 60 keV to obtain acceptable photon counts on the detector. For such an energy, the X-ray flux of the bending magnet beamlines of APS is approximately ten times lower than that at 20–25 keV (optimal energies). Moreover, all X-ray imaging components (scintillators, monochromator and mirror) become less efficient. The situation becomes even more difficult for larger samples, such as, for example, in the cases of primate brains.

Cutting large samples into smaller sections and scanning each section independently, followed by reconstruction and stitching procedures, is the only way to handle such samples. Minimizing the number of sample cuts is of great interest because each cut damages the sample structure and causes discontinuities among sections, thereby compromising the quality of segmentation and structures tracing. This can be of critical relevance such as for example in axons tracing to study the anatomy of neuronal pathways in normal and pathological states (Mizutani *et al.*, 2016 ▸; Foxley *et al.*, 2021 ▸; Wildenberg *et al.*, 2023 ▸).

**Figure 1:**
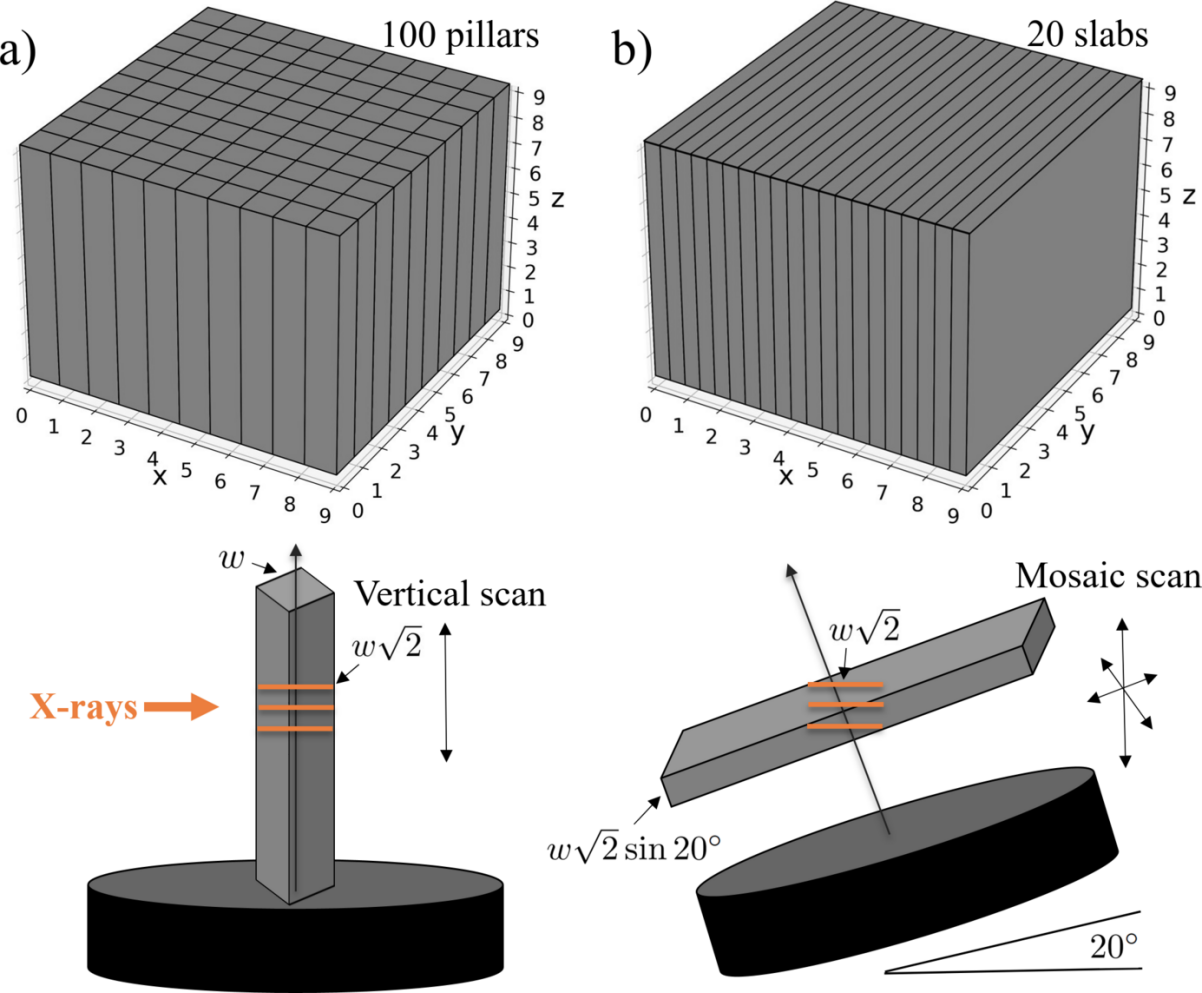
Schemes for scanning large samples by cutting them into parts with sufficient X-ray transmission: (*a*) tomography geometry with pillar-shaped parts, (*b*) laminography geometry with slab-shaped parts.

For regular tomography, samples are cut into pillars sufficiently thin to fit the experimental requirements. The pillars are then scanned at different vertical positions with overlaps. Reconstructions from the data acquired at these positions are then stitched to form the whole volume, see Fig. 1 ▸(*a*). Alternatively, the sample can be cut into slabs and scanned in the laminography geometry [Fig. 1 ▸(*b*)]. The whole slab is scanned at different horizontal and vertical positions with overlaps [mosaic scanning mode (Du *et al.*, 2018 ▸)]. The acquired data are then stitched together to form a big data volume for further laminography reconstruction. Following the sketches in the bottom part of Fig. 1 ▸, the slab thickness is chosen as 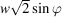 where *w* is the pillar width guaranteeing sufficient X-ray transmission along the maximum thickness 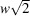, and φ denotes the laminography angle (φ = 20° in the figure). The optimal thickness can be found experimentally by analysing photon statistics on the detector and using the principles from Grodzins (1983 ▸) and Flannery *et al.* (1987 ▸). For the example in Fig. 1 ▸ with φ = 20° laminography angle, the total number of sections to be cut is approximately five times lower in the laminography case compared with tomography (100 versus 20); for φ = 30°, the number of slabs is seven times lower. Similar estimations for larger sample sizes reveal even greater improvements in the effectiveness of the laminography method, establishing it as a crucial X-ray imaging technique significantly minimizing sample damage.

## 3. Laminography setup at micro-CT beamline 2-BM

**Figure 2:**
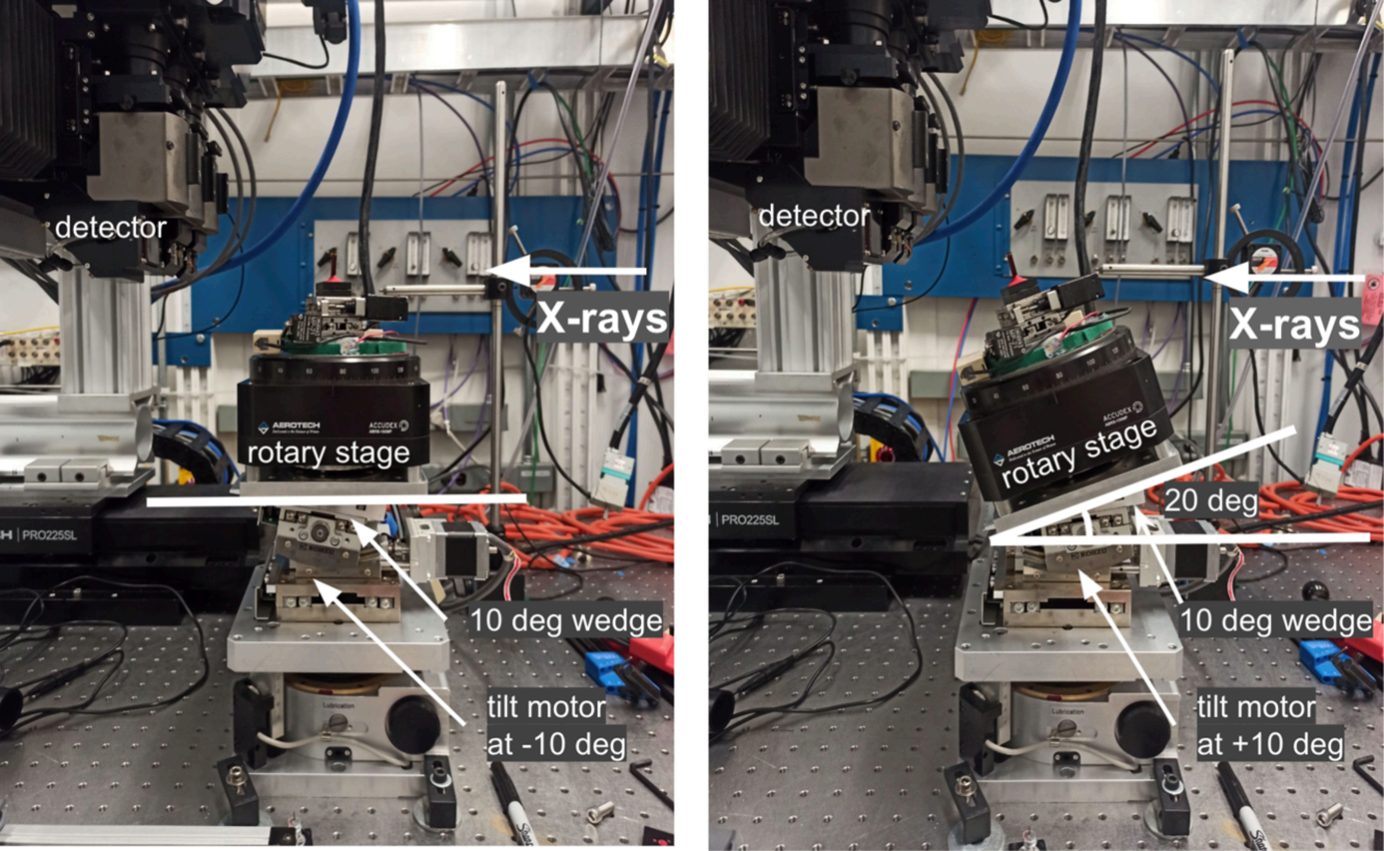
Sample stack with automatized switching between tomography (left) and laminography (right) geometries for conducting synchrotron experiments at sector 2-BM of the APS.

Most micro-CT beamlines at synchrotrons worldwide have similar setups for conducting experiments. They include a detection system, rotary stage and vertical/linear stages for alignment. Besides, often the setup includes tilt motors needed for adjusting the pitch (along the beam) and roll (orthogonal to the beam) angles to properly align the rotary stage for tomographic acquisition. The sample is placed on the top of the rotary stage, where a micro-positioning system is normally present for selecting the region of interest for scanning. An example of the sample stack motors implemented at beamline 2-BM of the APS is shown in Fig. 2 ▸.

In order to implement the laminography geometry for scanning, one needs to tilt the rotary stage at a significant angle with respect to the beam direction. Generally, the tilt motor located under the stage allows motion only for a limited range of angles because it is used to align the rotary stage parallel with respect to the beam. The direction of the beam is adjusted for instance after switching between the pink beam and monochromatic beam, or when changing the energy on the monochromator. This alignment correction is typically less than a couple of degrees. Most popular, compact and inexpensive tilt stages available on the market allow for travelling ±10 or ±15°. At 2-BM we use a Kohzu SA07A-R2L stage with ±10° travel range. Therefore, to achieve the 20° tilt angle required by laminography, we machined a 10° wedge and placed it under the rotary stage (see Fig. 2 ▸, right). The wedge does not need to have precise angular specification because the fine alignment can be achieved by adjusting the tilt/roll motors under the rotary stage and analysing the X-ray projections of a simple object like a tungsten pin. For instance, if the wedge is not perfectly flat then it is possible to compensate the misalignment using the motor that tilts the rotary stage orthogonal to the beam (roll alignment). Additional inaccuracies can be identified either through the use of a tungsten pin or by analysing the reconstructions.

Quick switching between tomography and laminography geometry during the beamline operation is important because, first, it reduces data acquisition delays during the switch over, and, second, it allows for more flexible data collection allowing to use the best geometry for the sample at hand and ultimately delivering higher quality 3D sample representation. In the current setup at 2-BM, the laminography geometry with 20° tilt angle is achieved by placing the stage to +10°, while positioning the stage angle to −10° gives us 0° tilt against the beam, *i.e.* the regular tomography geometry. It is worth mentioning that this quick switch between geometries also makes the alignment procedures easier. For instance, procedures such as the rotation axis alignment and adjusting the roll stage angle (tilt orthogonal to the beam) can be first done in the tomography geometry and then reused in laminography. The misalignment issues can also be resolved in the laminographic reconstruction process, as will be shown in the next section.

In the alignment procedures outlined above, it is assumed that the plane containing the moving trajectory of the pitch and the X-ray beam are parallel. This parallelism remains unaffected by the addition of any form of wedge and can be verified using a standard setup without the wedge. If the pitch trajectory is not parallel to the beam, the angle between the two can be determined using a tungsten pin. This angle serves as a fixed reference and remains unchanged even after the wedge is added. Subsequently, it can be utilized in the reconstruction procedures.

**Figure 3:**
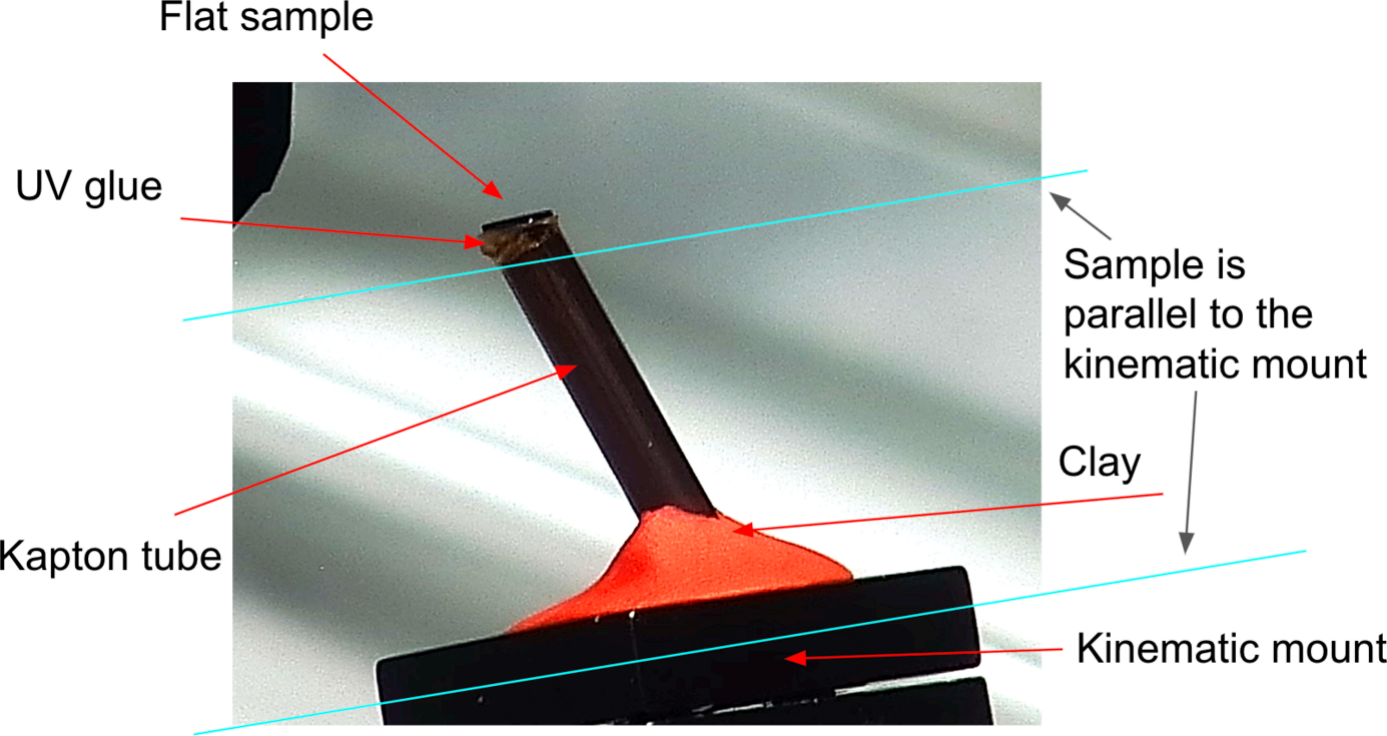
An example of sample mounting for laminography data acquisition.

Another important aspect of our laminography implementation is the sample mounting procedure. Regular mounting strategies, such as glueing to a pin or fixing in a holder, are not applicable because the pin or holder will block the beam and will not allow informative projections to be captured for many regions. Instead, we propose using Kapton tubes that are semi-transparent to X-rays (see Fig. 3 ▸). Kapton tubes with 200 µm wall thickness are stiff enough to keep the sample stable during rotation, and do not significantly attenuate the X-ray beam reaching the detector when working with hard X-rays (>10 keV). The diameter of the tube, as well as the wall thickness, can be chosen based on the sample shape and weight. Flat samples are glued to one side of the tube with epoxy or with a UV glue supplied for instance by Bondic. In our experience, the UV glue is less radiation sensitive and more transparent to X-rays than epoxy. Moreover, the glue is much easier to use since it does not have any timing requirements for mounting. While the sample is glued to one side of the Kapton tube, another side of the tube is attached to a kinematic mount with clay. Alternatively, one could also use the UV glue for this. For more efficient imaging, flat samples should be mounted parallel to the kinematic mount, otherwise the X-ray propagation distance through the sample may be significantly increased for some angles, resulting in potential beam blockage.

## 4. Laminography reconstruction

In this section, we will formulate the laminography reconstruction problem in terms of operators and discuss methods for fast evaluation of these operators.

The forward laminography operator, or laminographic projection, maps a 3D object attenuation function μ(*x*_1_, *x*_2_, *x*_3_) to data *d*(θ, *u*, *v*), where *u*, *v* are detector coordinates, and θ is the rotation angle. In this work we define the laminography tilt angle φ as the angle between the rotation axis and beam direction (horizontal). Note that in some literature this angle is measured between the rotation axis and the axis orthogonal to the beam (vertical). For this, variable φ should be changed to 90 − φ in all further formulas. We defined the laminography projection as follows, 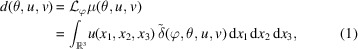
where 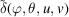 is a multiplication of two delta function defining line directions, 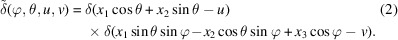
The measured signal on the detector is linked to the intensity transmitted through the sample (following the Beer–Lambert law) and also includes contributions from the dark field *d*_d_(*u*, *v*) (image on the detector when the beam is off) and flat field *d*_f_(*u*, *v*) (image on the detector when the beam is on and the sample is out), 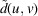 = 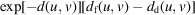 + 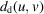. Therefore, before solving the inverse problem for (1), the dark/flat-field correction and taking the negative logarithm procedures are applied to the raw detector data. Note that for φ = 0, the integral in (1) becomes a general Radon transform used in tomography.

The inversion formula is given by means of filtered backprojection (FBP), 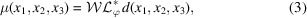
where operator 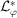 is adjoint to 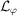, called laminographic backprojection, and written as 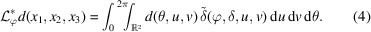
The operator 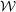 is described as a convolution with a transfer function being a suitable scaled version of 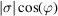 (ramp filter), where σ denotes the conjugate variable of *u*. Similar to regular tomography, instead of the ramp filter it is common to consider low-pass filters (Shepp–Logan, parzen) for decreasing noise in reconstructions.

Direct discretization of line integrals for evaluating the forward and adjoint laminography operators (1) and (4) with linear interpolation has computational complexity 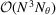 if we assume that reconstruction is made on a *N* × *N* × *N* volume, the detector size is *N* × *N*, and the number of rotation angles is *N*_θ_.

Alternatively, formulas (1) and (4) can be evaluated with Fourier-based methods of lower computation complexity (Myagotin *et al.*, 2013 ▸; Voropaev *et al.*, 2016 ▸). In this work, we define the Fourier transform as 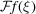 = 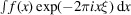 and use subscripts with operation 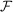 to specify the transform dimensions or the grids it acts to. Using the Fourier transform properties of the delta function, it can be readily verified that 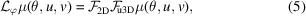
where 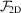 is a regular two-dimensional Fourier transform that in a discrete case is computed between equally spaced grids (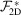 – the adjoint/inverse transform). Operator 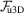 denotes the three-dimensional Fourier transform that in a discrete case is applied from the equally spaced grid (*x*_1_, *x*_2_, *x*_3_) to an unequally spaced grid (ξ_1_, ξ_2_, ξ_3_) with 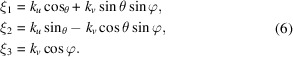
By making use of properties of the Fourier transform, the adjoint laminography operator can be calculated by replacing the 2D and 3D Fourier transforms with their adjoints and with reversing the operators order, namely, 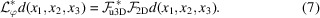
Computational complexity for discrete evaluating the forward and adjoint laminography operators by (5) and (7) in terms of FFT is lower than by using direct discretization of line integrals in (1) and (4). Indeed, the two-dimensional Fourier transform between equally spaced grids in both formulas is directly computed by means of FFT. For the three-dimensional Fourier transform 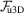 there also exist fast methods based on unequally spaced fast Fourier transform (USFFT) (Dutt & Rokhlin, 1993 ▸; Beylkin, 1998 ▸). In short, the methods utilize a Gaussian function ψ exhibiting certain properties, to rewrite the transform in the form of convolution, 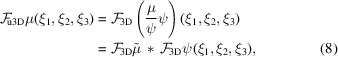
where 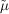 = 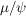. For the discrete version, the Fourier transform 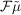 is calculated on an equally spaced grid, and the convolution allows switching to unequally spaced coordinates. The whole 3D USFFT procedure for computing the Fourier transform from equally to unequally spaced grid is described by the following steps:

(i) Division by ψ in the space domain.

(ii) 3D FFT.

(iii) Convolution-type operation in the frequency domain.

The adjoint laminography operator is computed with the inverse 3D USFFT from the unequally to equally spaced grid. For that, the steps above should be done in reverse order, and the second step is replaced by ‘inverse 3D FFT’. The resulting computational complexity for evaluation of the forward/adjoint laminography operator is given by the complexity of the 3D FFT, *i.e.*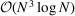. Clearly, the Fourier-based method is computationally more favourable than the direct discretization of the line integral. However, for small data sizes and if the number of projection angles (*N*_θ_) is very small then the direct discretization may work faster due to the code implementation.

**Figure 4:**
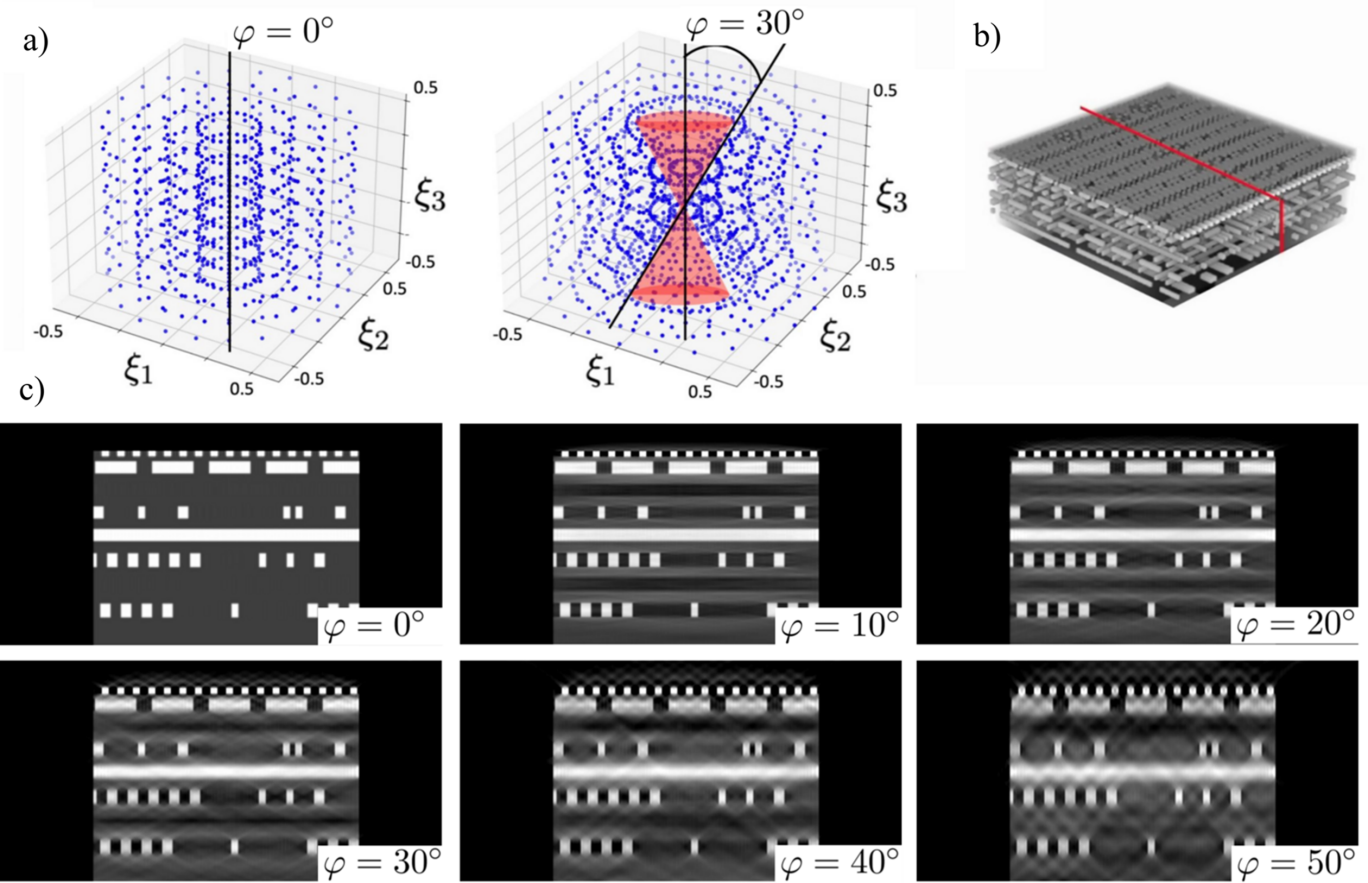
Undersampling problem in laminography: (*a*) grids (ξ_1_, ξ_2_, ξ_3_) in the Fourier space defined in (6) for φ = 0° (regular tomography) and for φ = 30° showing the missing cone in red; (*b*) synthetic integrated circuit dataset with the red-coloured position of the vertical slice for demonstrating reconstruction quality, (*c*) examples of vertical slice reconstruction in laminography for different tilt angles.

Formulation of the reconstruction problem with the Fourier-based method can be used to demonstrate the general laminography undersampling problem, *i.e.* the missing cone in the Fourier space, see Fig. 4 ▸(*a*). The figure depicts grids (ξ_1_, ξ_2_, ξ_3_) in the Fourier space for laminography angles φ = 0° (regular tomography) and for φ = 30° where the region marked with a red cone corresponds to missing information. To demonstrate the effect of the missing cone on reconstruction we generated laminography data for a synthetic integrated circuit data set by using formula (5) and reconstructed it by using formulas (3) and (7). Fig. 4 ▸(*b*) shows a 3D volume rendering of the integrated circuit, and an approximate red-coloured position of the reconstructed slice used for quality comparisons. Fig. 4 ▸(*c*) shows the reconstructed slice for the tilt angles 0°, 10°, 20°, 30°, 40° and 50°. The ‘tail’ artefacts along the angular span are clearly visible, especially for 40° and 50° tilt angles. We also observe that most of the visible artefacts originate from the sample features having higher amplitudes (metal layers of white colour) and propagate to the regions of uniform intensity. Therefore, it can be inferred that better quality laminography results are obtained with lower tilt angles (≤30°) and for more homogeneous samples.

**Figure 5:**
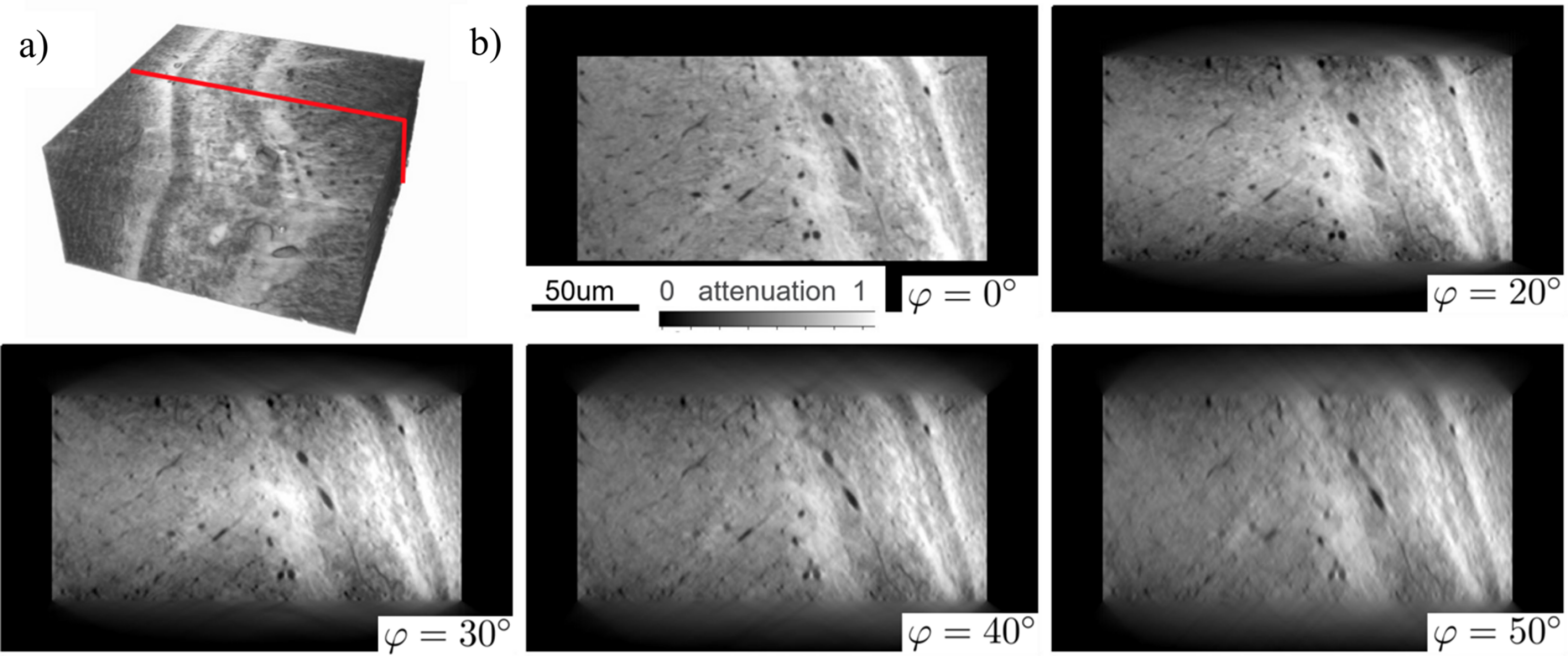
Reconstruction of a semi-synthetic brain dataset: (*a*) a cropped region of the brain dataset used for data modelling – the red colour shows the position of the vertical slice for demonstrating reconstruction quality; (*b*) examples of vertical slice reconstruction in laminography for different tilt angles.

In Fig. 5 ▸ we validate the influence of these artefacts in a realistic case of scanning a section of osmium-stained mouse brain. To demonstrate reconstruction of such a sample with different laminography angles, we formed a ‘semi-synthetic’ mouse brain dataset based on high-quality data purposely acquired in the computed tomography geometry. The reconstruction was then cropped to a small slab shape and used as an initial object for generating laminography data for different angles, where forward laminography operator (1) was used to generate projections. The figure shows that mouse brain features are not significantly affected by the artefacts for angles φ ≤ 30°, except probably a small amplitude loose at the top and bottom parts of the reconstructions. In the results with φ = 40°, 50°, the degradation of fine brain features becomes visible.

The integrated circuit and brain volumes had sizes (256, 256, 128); projection data were generated for 384 angles over a 360° range for a simulated detector with sizes (256, 256). It is worth noting that in contrast to regular tomography operating with data from a half-circle angular range (180°), the laminography geometry requires angles from the whole circle to properly fill the frequency space for reconstruction.

For suppressing laminography artefacts typical for the samples like the synthetic integrated circuit above, one can use reconstruction with total variation regularization, as was demonstrated by Fisher *et al.* (2019 ▸). In Appendix *A*[App appa], we formulate the reconstruction problem with regularization, solve it by employing the proposed implementation of the laminography operators, and demonstrate enhancement of integrated circuit reconstruction results for large laminography angles.

## 5. GPU acceleration of reconstruction

Nvidia GPUs are commonly used for tomography reconstruction since they demonstrate more than ten times acceleration compared with CPU-based implementations (Andersson *et al.*, 2016 ▸; Nikitin, 2023 ▸). In tomography, each data slice (sinogram) can be processed independently to obtain a horizontal slice through a 3D reconstructed object. Modern GPUs have enough VRAM memory to process sinograms of sizes more than 30k × 30k pixels (Nikitin, 2023 ▸). In laminography though, reconstruction of one slice through the object requires data from different sinograms, making the GPU memory requirements more demanding. Therefore, efficient data chunking, as well as CPU-GPU data transfer protocols, need to be developed.

For direct discretization of the line integral in the backprojection formula (4), the chunking can be done in the slice *x*_3_ and angle θ directions. A reconstructed chunk of slices in *x*_3_ is obtained by summing reconstructions from all individual chunks of angles. The GPU memory requirements are defined based on discretizing 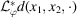 = 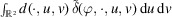, which does not involve operations on 3D arrays and therefore can be computed by chunks that fit the GPU memory.

Reconstruction with the Fourier-based method for evaluating the backprojection by formula (7) involves operations on 3D arrays. For instance, computing 3D Fourier transforms on unequally spaced grids involves computing 3D FFTs and 3D interpolation-like procedures in the frequency domain. Implementing such procedures on a GPU even for a 2048^3^ dataset requires more than 64 GB memory, which is beyond the capability of most modern GPUs. Therefore, we decompose the unequally spaced 3D Fourier transform into a combination of batched 1D and 2D transforms by splitting variables in the Fourier integral as follows, 
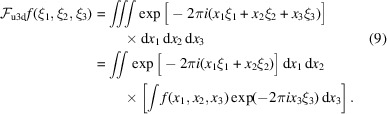
It turns out that computing 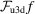 can be done in two steps: 1D USFFT with respect to variable *x*_3_, followed by 2D USFFT with respect to variables *x*_1_, *x*_2_. Data chunking to fit GPU memory is done by columns and slices, respectively.

**Figure 6:**
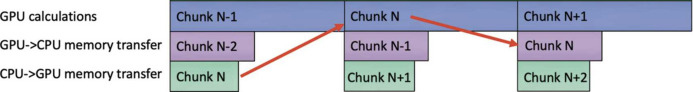
A scheme for asynchronous data processing by chunks where GPU reconstructions are overlapped with data transfers.

GPU data processing by chunks involves three operations: CPU to GPU data transfer, computations on GPU, and GPU to CPU data transfer. In the aforementioned approach with batched processing the CPU–GPU data transfer takes a significant amount of total computation time for reconstruction. Therefore, in this work we adopt the approach proposed by Nikitin (2023 ▸) and organize an efficient pipeline for asynchronous data processing by chunks. Schematically the pipeline is shown in Fig. 6 ▸. With this pipeline, three operations are executed simultaneously: CPU–GPU memory transfer for Chunk *N*; GPU computations for Chunk *N* − 1; and GPU–CPU memory transfer for Chunk *N* − 2. We used this approach in all steps that involve chunking for computing backprojection (4), *i.e.* 2D USFFT, 2D FFT and 1D USFFT.

Asynchronous execution of computations and fast data transfers is implemented in Python with the *CuPy* interface. The *CuPy* interface allows for creating Nvidia CUDA Streams and allocating pinned memory needed to overlap computations and data transfers. To implement the overlap, the pinned memory on CPU and device memory on GPU should be both allocated for two input data chunks and two output chunks. Three CUDA streams run simultaneously by switching between chunks: the first stream performs a data copy to the first input chunk of the pinned memory, followed by transfer to the first input chunk of GPU memory. The second stream performs GPU computations on the second input chunk in GPU memory (whenever it is available) and places the result in the second output chunk in GPU memory. The third stream executes a data transfer from the first output chunk in GPU memory to the first output pinned memory chunk. The chunk is then copied to a corresponding place in the resulting array. After processing each chunk, all streams synchronize and switch the chunk ID (0 or 1) they operate with.

**Figure 7:**
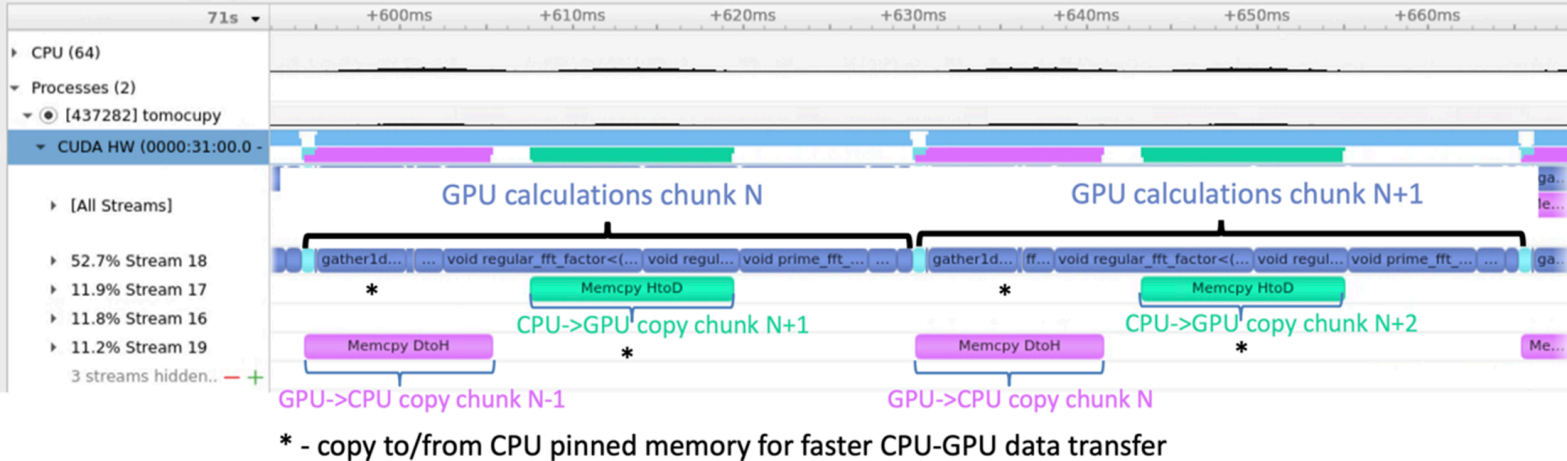
Timeline view report from the Nvidia Nsight System tool for asynchronous execution of the 1D USFFT operation for computing the laminographic backprojection operator.

Asynchronous execution can be verified with the Nvidia Nsight System profiling tool (https://developer.Nvidia.com/nsight-systems). As an example, in Fig. 7 ▸ we demonstrate profiling results after executing a batch of 1D USFFT. Memory transfers take more than 70% of time for GPU calculations; however, they are done asynchronously and therefore do not affect performance. Note that, in comparison with the schematic in Fig. 6 ▸, each CPU to GPU data transfer (green block) starts later than GPU data processing (dark blue block). This is due to the fact that some CPU time is spent transferring data to pinned memory, which is referred to CPU execution and is not shown in the profiler. Similarly, some CPU time for operation with pinned memory is spent after transferring data from GPU to CPU. In Fig. 7 ▸ we marked these blocks with an asterisk (*) for clarity.

In Table 1 ▸ we demonstrate performance tests of the laminographic reconstruction implemented in the *TomocuPy* package. Besides reconstruction with the Fourier-based method (*Fourierrec*) and by direct discretization of the backprojection line integral (4) (*Linerec*), the table also shows time for data pre-processing and read/write operations with an SSD storage. The pre-processing step includes dark-flat field correction, ring removal (Vo *et al.*, 2018 ▸) and phase retrieval by Paganin filtering (Paganin *et al.*, 2002 ▸). All operations are implemented in *TomocuPy* using a similar GPU pipeline approach as for the backprojection.

The tests were performed using synthetic HDF5 format datasets of different sizes. The datasets were generated for *N* 8-bit laminographic projections with *N* × *N* detector sizes, where *N* ranges from 1024 to 4096. The laminography tilt angle was set to 20°, although this angle does not affect the performance significantly. Reconstructed volumes (*N* × *N* × *N*) were obtained as sets of tiff files in 32-bit precision. Chunk sizes in projection angles and reconstructed slices were chosen by taking into account the GPU memory limitation and overall performance. For instance, for *N* = 1024 the chunk size in angles was 128, while for *N* = 4096 the size was 4. The chunk sizes as well as other reconstructions are given through the *TomocuPy* command-line interface – see the next section for more details.

Performance tests were carried out on a machine with Intel Xeon Gold 6326 CPU @ 2.90 GHz, 2 TB DDR4 3200 memory, one Nvidia Tesla A100 with 40 GB memory, and Intel SSD D7-P5510 Series PCIe 4.0 NVMe disks. Installed software included Python 3.9, CuPy 12.1, Nvidia CUDA toolkit 12.1.

Table 1 ▸ shows that the *Fourierrec* method significantly outperforms *Linerec* because of more favourable computational complexity [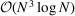 versus 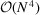]. For *N* = 1024 the acceleration is about three times, while for *N* = 4096 it is higher than 30 times. Lower computational complexity is crucial in developing new reconstruction algorithms because detector sizes become bigger. Even if an algorithm with 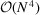 complexity is accelerated with large computational resources like multiple GPUs, for a large enough *N* it will become slower than the one with 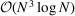 complexity. From the table, we see that increasing data sizes by a factor of two (*e.g.* 2048 → 4096) gives the difference between reconstruction times as 2^4^ = 16 for the *Linerec* method and 2^3^ = 8 for the *Fourierrec* method. However, we also observe that increasing data sizes by a factor of 1.5 (*e.g.* 3072 → 4096) does not give 1.5^3^ ≃ 3.4 time difference (77.7 s versus 164 s). This can be explained by the fact that the FFT procedure on GPU is very well optimized for the sizes that are powers of two, making processing data for *N* = 4096 more optimal. A simple test of the two-dimensional FFT operation on GPU gives execution times of 0.17 ms, 0.43 ms and 0.5 ms for sizes 2048 × 2048, 3072 × 3072 and 4096 × 4096, respectively, which confirms the slowdown for the sizes that are not powers of two.

Another observation from Table 1 ▸ is the relatively high performance of the read and write operations. In these tests we utilized PCIe 4.0 NVMe SSDs that allow parallel operations with the storage using multi-threading. With this system we were able to reach up to 3 GB s^−1^ for reading and writing. Regular HDD storage is more than five times slower and therefore may become a bottleneck for reconstruction. Therefore we note that, besides powerful GPUs, the NVMe SSD storage is also a crucial component for accelerating the whole reconstruction process. The table demonstrates results for up to 4096 × 4096 × 4096 reconstructed volumes, which corresponds to 256 GB of RAM. For bigger sizes the data may not fit into RAM and it will be necessary to operate with data chunks by communicating with the hard disk for each pre-processing procedure and reconstruction step (*e.g.* Paganin Filter, USFFT1D, USFFT2D, *etc*.). In this case a fast SSD storage could be a good alternative to RAM.

## 6. Application to neuroimaging

**Figure 8:**
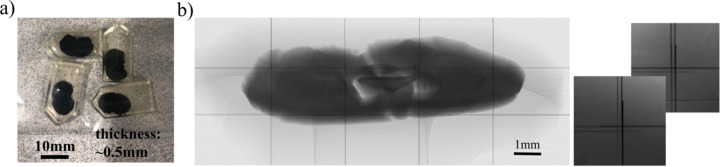
Mouse brain samples stained with heavy materials [osmium tetroxide, uranyl acetate and lead (II) nitrate] for laminographic scanning (*a*), and an example of laminographic projection after data stitching and dark-flat field correction (*b*). The right part of (*b*) shows zoomed-in stitched regions of adjacent datasets, with black lines indicating the datasets borders after automatic stitching.

To demonstrate the applicability of the developed laminographic implementation for imaging large samples we considered imaging of four sequential sample slabs cut from a whole mouse brain, see Fig. 8 ▸(*a*).

The slabs were prepared using protocols for electron microscopy. Briefly, a mouse is transcardially perfused with aldehyde fixatives and sectioned into ∼500 µm coronal sections. Sections are then stained with heavy metals [*i.e.* osmium tetroxide, uranyl acetate and lead (II) nitrate] (Hua *et al.*, 2015 ▸) to increase the X-ray absorption contrast. After staining, the samples were dehydrated and embedded in an epoxy resin to make it more X-ray resistant. Each slab has approximate sizes of 12 mm  ×  8 mm, with a thickness of about 500 µm. For 25 keV energy (optimal for 2-BM beamline of APS) such a thickness indicates satisfactory X-ray transmission, ranging from 15% to 30% depending on the sample rotation angle. The sample slabs were glued to a Kapton tube which in turn was attached to a kinematic mount with the Bondic UV glue (see Fig. 3 ▸). Experiments were conducted in the downstream experimental station located at 50 m from the source. For the measurements we used a filtered pink X-ray beam with an energy peak at 25 keV. A 8 mm glass filter was placed upstream of the sample to cut low X-ray energies and decrease the radiation damage. The exposure time per projection was 50 ms. An ORX-10G-310S9M camera with 6464 × 4852 pixels (pixel size 3.45 µm × 3.45 µm) recorded projections from a 25 µm-thick GGG:Eu scintillator, magnified through a 7.5× lens yielding a resulting isometric voxel size of 0.92 µm after 2 × 2 binning. We experimentally chose 200 mm as the distance between the sample and the objective to increase propagation-based phase contrast. The detector field of view after binning was also cropped to the size 3232 × 2256 due to the beam shape. Detector data were collected in 12-bit mode and stored as 16-bit images. The laminography tilt angle was set to 20° by adding the 10° wedge under the rotary stage and setting the tilt motor to 10° (see Fig. 2 ▸).

The mouse brain slabs were scanned in the mosaic scanning mode by moving the whole sample stack by five and three steps in the horizontal (orthogonal to the beam) and vertical and directions, respectively. Because of low repeatability and accuracy of the vertical and horizontal motors under the rotary stage, an overlap of 300–400 pixels between projections of two adjacent datasets was set to perform image registration for more precise image stitching. For feature-based image registration we used the SIFT algorithm (Lowe, 2004 ▸). Data from each two overlapped regions were summed with linearly changing weights in the range between 0 and 1. An example of one of the stitched projections after dark- and flat-field correction is shown in Fig. 8 ▸(*b*). Black lines indicate borders between different datasets used for stitching. Projection size after stitching is 14960 × 5936 pixels.

Fifteen thousand tomographic projections were collected in a fly scan mode while the sample was continuously rotated over 360° at 0.48° s^−1^, which together with collection of dark and flat fields yielded 13 min total acquisition time per dataset, 5 × 3 × 13 = 195 min per a slab, and 4 × 5 × 3 × 13 = 780 min (13 h) for scanning all four slabs. The total size of the acquired raw data was about 12 TB, after projections stitching it reduced to 10 TB.

For reconstruction we used the FBP formula (3) with the backprojection operator implemented with the proposed Fourier-based method, see formula (7). The iterative reconstruction was not considered since laminography artefacts for 20° tilt angle are not significant for such kinds of samples (see tests in Fig. 5 ▸). Because of the huge data sizes, reconstruction was done by steps with saving and loading intermediate results for chunked data processing.

To accelerate reconstruction, we utilized several nodes of the Polaris supercomputer of the Argonne Leadership Computing Facility (https://www.alcf.anl.gov/polaris). Each Polaris node is equipped with an AMD EPYC Milan processor and four Tesla A100 GPUs with the SXM connection interface and high-speed HBM memory architecture. The storage called Eagle is based on a Lustre file system residing on an HPE ClusterStor E1000 platform equipped with 100 PB of usable capacity across 8480 disk drives. This ClusterStor platform also provides 160 object storage targets and 40 metadata targets with an aggregate data transfer rate of 650 GB s^−1^.

### 6.1. Reconstruction pipeline with *TomocuPy* calls

Before demonstrating reconstruction of large mouse brain slabs, we will describe our proposed laminographic pipeline for manual adjustments of the rotation axis and laminographic tilt angle by using a dataset acquired for a small mouse brain slab that almost fits the detector field of view. The dataset consists of 3000 projections of size 3232 × 2256.

In the proposed laminography implementation, the tilt angle is not given exactly since the wedge is manufactured with low angle accuracy. Moreover, the wedge may have an error in the roll angle, *i.e.* the one in the direction orthogonal to the beam. The roll angle issue can be resolved with the regular tomographic setup: by moving the tilt angle to 0°, rotating the camera or the roll motor under the rotary stage, moving the tilt angle back to 20°.

**Figure 9:**
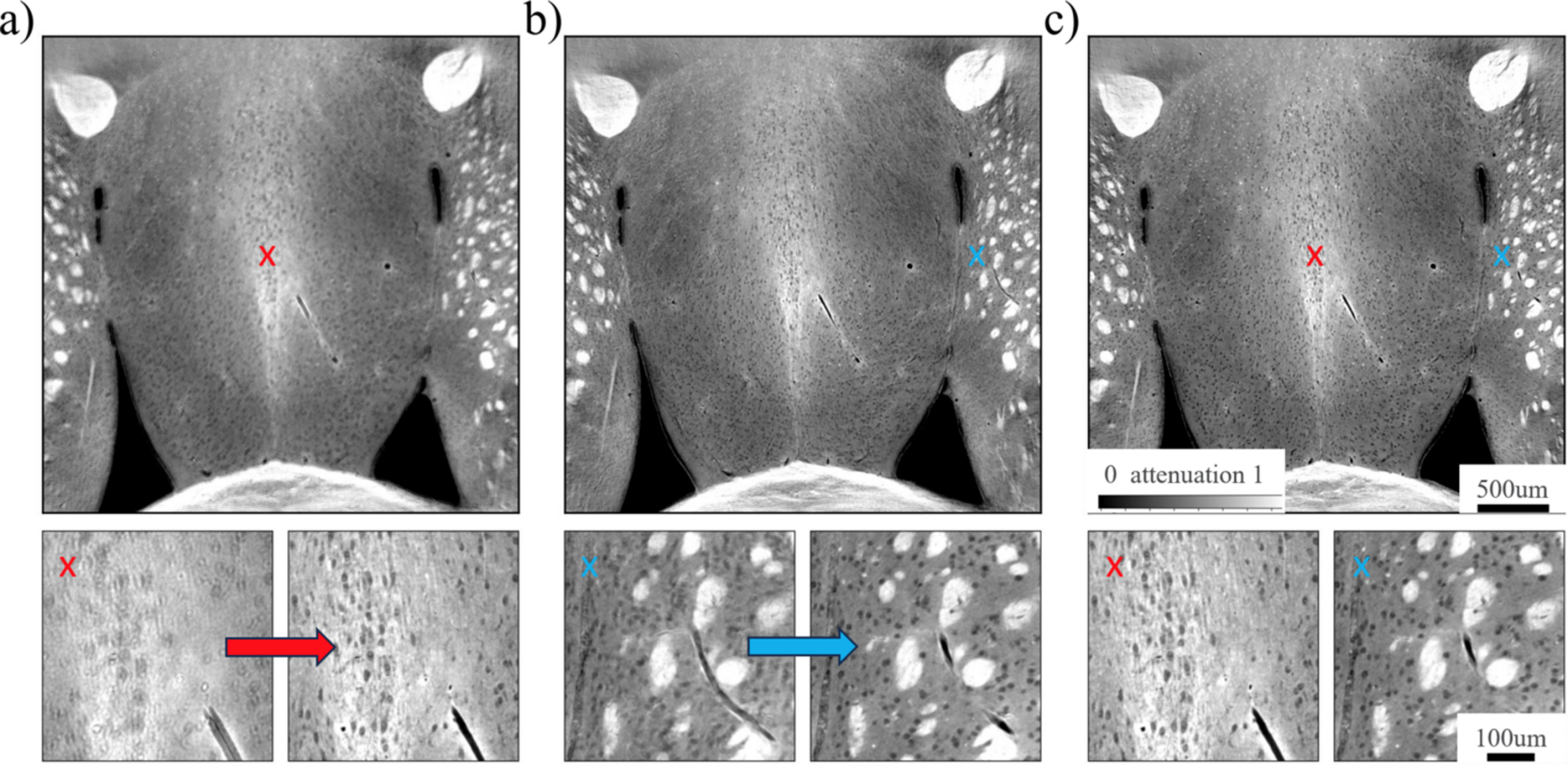
Reconstruction strategy for the low-cost laminography: (*a*) Step 1, searching the rotation axis by examining the middle part of the image, (*b*) Step 2, searching the laminography tilt angle by setting the rotation axis from Step 1 and examining the border part of the image, (*c*) Step 3, full reconstruction with chosen rotation axis and laminography tilt on Steps 1 and 2.

Searching for the laminography tilt angle and searching for the rotation axis can be performed during reconstruction. We propose the following strategy:

Step 1. Choose an approximate value for the laminography tilt angle and for the rotation axis, and run a reconstruction of one slice for different rotation axes. In the *TomocuPy* command-line interface the command should include the parameter 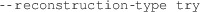 and is executed as: [Chem scheme1]
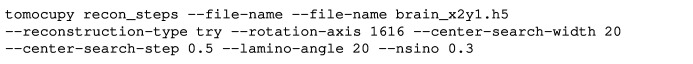
The command generates reconstructions of one slice for rotation axes [1616 − 20,…, 1616 + 20) and for 20° laminography tilt angle. Reconstruction of the middle part of images is not influenced much by the error in the laminography tilt angle, therefore the rotation axis can be found by scrolling through the images and examining only the middle part of them, see Fig. 9 ▸(*a*).

Step 2. Choose an approximate value for the laminography tilt angle, set the rotation axis found in Step 1 and run a reconstruction of one slice for different laminography tilt angles. In *TomocuPy* command-line interface the command should include the parameter 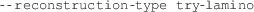 and may be executed as: [Chem scheme2]
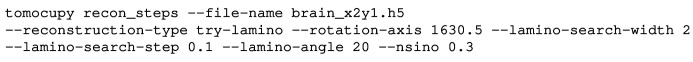
The command generates reconstructions of one slice for rotation axis 1630.5 and for [20 − 2,…, 20 + 2)° laminography tilt angles. Reconstruction of the border parts of the images is influenced by the error in the laminography tilt angle, therefore the angle can be found by scrolling through the images and examining their border parts, see Fig. 9 ▸(*b*).

Step 3. Use the rotation axis found in Step 1 and the laminography tilt angle from Step 2 to run a reconstruction of the full volume with setting parameter 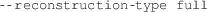:[Chem scheme3]
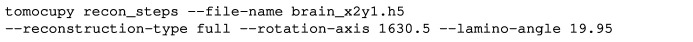
The command generates a reconstruction of the full volume, see Fig. 9 ▸(*c*).

An additional phase-retrieval procedure with the Paganin filter is performed by adding 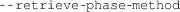, 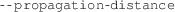, …, parameters; for details see the *TomocuPy* documentation.

**Figure 10:**
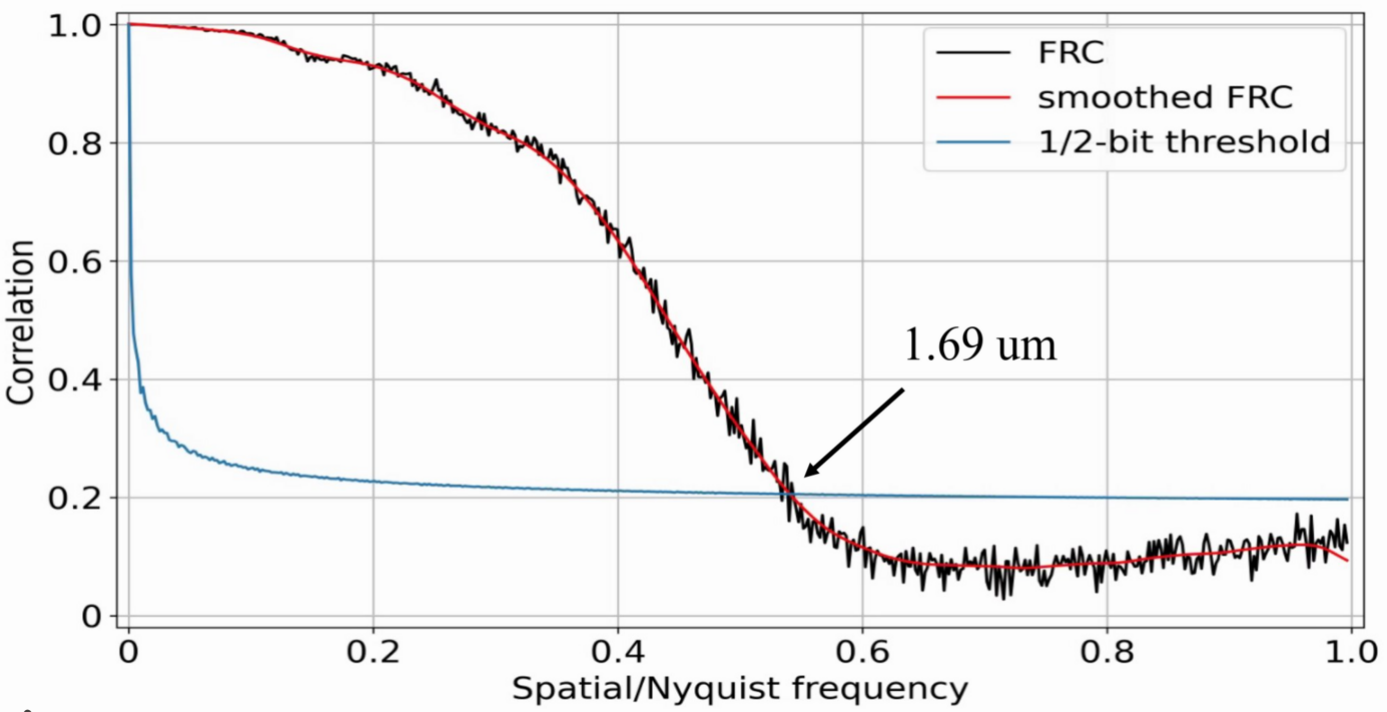
Resolution estimation by Fourier ring correlation with the 1/2-bit criterion.

Resolution levels were estimated by computing the Fourier ring correlation (FRC) (Van Heel & Schatz, 2005 ▸) between reconstructions obtained from two independent sets of 3000 projections. We used the 1/2-bit resolution criterion. Since the reconstructed volume is thin, the resolution levels were estimated by slices and the plot with the lowest resolution (higher level in micrometres) was chosen as the final result, see Fig. 10 ▸. The intersection between the lines for the 1/2-bit criterion and the FRC corresponds to 1.69 µm resolution estimation.

### 6.2. Results for the large mouse brain data

**Figure 11:**
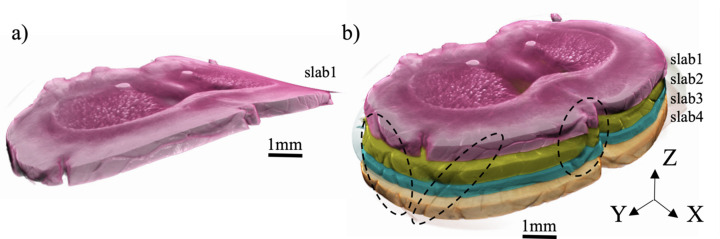
3D visualization of reconstructed mouse brain slabs: (*a*) initial bent reconstruction of the slab 1 (top sample part), (*b*) four straighten slab volumes stitched together.

In the following we will demonstrate reconstruction of full brain slabs where data for each slab has sizes 15000 × 14960 × 5936 in 16-bit precision. Fig. 11 ▸ shows 3D volume rendering of reconstructed slabs in ORS Dragonfly package after binning reconstruction by a factor of eight in each dimension. The brain slabs were bent and tilted during the sample preparation, see Fig. 11 ▸(*a*). Therefore we used additional postprocessing procedures to straighten reconstructed volumes. The images were rotated and unbent with *Image.rotate()* and *Image.distort()* methods from the *Ward* Python package. After straightening the slabs they were stitched together, see Fig. 11 ▸(*b*). Black dashed ellipses in the figure show matching features between adjacent slabs.

**Figure 12:**
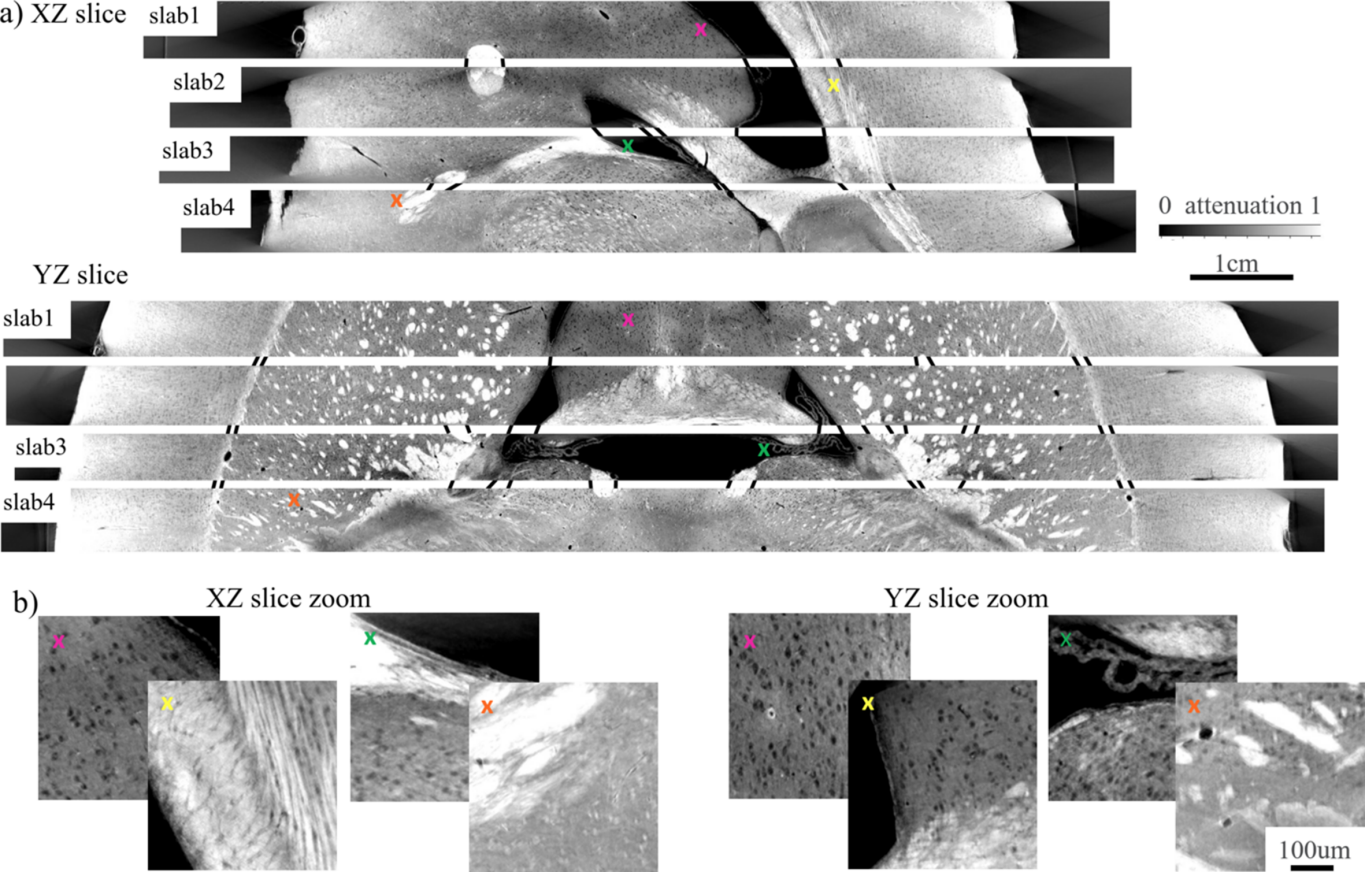
(*a*) Stitching reconstructed mouse brain slabs in vertical directions *XZ* and *YZ*. (*b*) Corresponding zoomed-in regions marked with coloured crosses.

**Figure 13:**
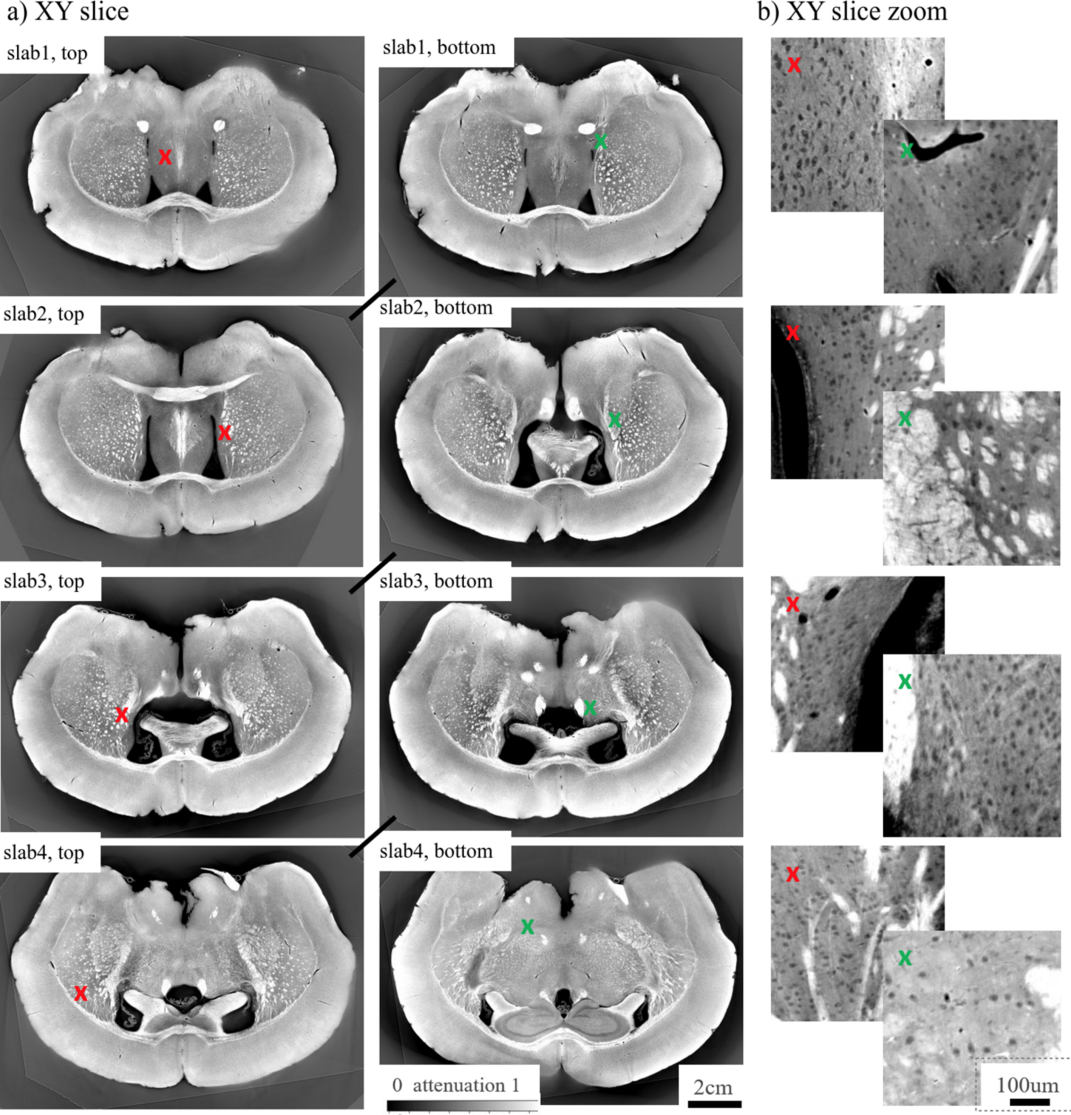
(*a*) Top and bottom slices in the horizontal direction *XY* for each slab. (*b*) Corresponding zoomed-in regions marked with coloured crosses.

Figs. 12 ▸ and 13 ▸ show reconstructed slices through the whole 3D volumes in high resolution. *XZ*, *YZ* and *XY* directions for the slices are defined based on the axes depicted in the bottom part of Fig. 11 ▸. We carried out a visual inspection of slices for different slabs and found similar features that can be used for stitching. Black lines between the slices in Fig. 12 ▸(*a*) show possible connections between the features. Accurate stitching is not possible because parts of the sample were destroyed/bent due to the cutting procedure. Based on the reconstructions, we can assume that 100 µm-thick layers between slabs were destroyed while cutting. It should be also noted that the brain was cut before embedding with petropoxy. This procedure may also affect the slab structures. The missing layer due to cutting can be also observed by comparing top and bottom slices in the *XY* direction in Fig. 13 ▸(*a*). The slices that should look similar are connected with black lines. For instance, ‘slab1, bottom’ and ‘slab2, top’ have similar features, although they are not very close to each other. Some parts of the top and bottom slices are blurred, see for instance the bottom right part of ‘slab1, top’, or the top right of ‘slab3, bottom’. This is due to the bent structure of the slabs. Although the images were straightened after reconstruction using the *Ward* Python package, local deformation is not easily compensated. The imaging quality can be analysed using the zoomed-in regions demonstrated in Figs. 12 ▸(*b*) and 13 ▸(*b*). The regions are taken at the positions indicated by coloured crosses in the whole slab images. The obtained imaging resolution allows for segmenting axons (black dots) in most places.

Resolution levels were estimated with the FRC as it was shown earlier for a small mouse brain sample. The middle part of the sample confirms 1.6–1.7 µm resolution. The levels on the sample borders are lower due to radiation damage. Iterative approaches with compensating sample deformation, *e.g.* the one from Nikitin *et al.* (2021 ▸), may be further considered to improve image quality and resolution.

## 7. Conclusions and outlook

The proposed laminographic scanning strategy, coupled with an innovative laminography instrument setup at 2BM, APS, and advanced reconstruction capabilities integrated into the *TomocuPy* package, not only facilitates the scanning of flat-shaped samples but also showcases the potential for imaging larger samples with minimal cutting procedures. As a primary illustration, we focused on imaging four sequential slabs from an entire mouse brain sample with a measured resolution of 1.69 µm (0.92 µm voxel size). This approach allowed us to trace connections between the slabs and discern axons in high-resolution reconstructions.

While the laminography imaging technique is already established at the bending-magnet beamline 2-BM of the APS, our work suggests several avenues for significant improvement in brain imaging quality. First and foremost, the development of more refined cutting mechanisms is imperative. In our current sample preparation, the missing layer destroyed during cutting was approximately 100 µm, complicating accurate axon tracing or making it impossible in some instances. One can potentially consider methods used in electron microscopy where the destroyed layer could be less than a micrometre (Mikula & Denk, 2015 ▸). Another possible method involves the development of methods of sectioning the brain prior to staining with heavy metals. Indeed, we have recently shown that a new machine used for sectioning aldehyde-fixed brains, called the Compresstome, has an estimated tissue loss between sections of approximately 680 nm (Wildenberg *et al.*, 2023 ▸). Given that ∼680 nm is near the size of a single pixel in our measurements, it is possible that the loss could be even smaller. Such an approach also offers the advantage that staining whole brains with heavy metals is difficult due to their poor diffusion, and protocols have only been demonstrated on whole mouse brains. Sectioning the tissue first and then staining it for X-ray imaging would bypass this limitation and pave a pathway towards imaging arbitrarily large brains.

Addressing the issue of the cut section deformations presents another potential enhancement. This can be approached by either cutting into slabs after embedding the entire sample in petropoxy or by considering more advanced methods for straightening the slabs. For example, the warp filtering method proposed by Ju *et al.* (2006 ▸) for dealing with wavy histological mouse brain sections in optical microscopy can potentially be adapted for 3D X-ray images.

The challenge of projection stitching for mouse brain data arises from low contrast, leading to insufficient features for accurate alignment. To overcome this, future efforts will involve the incorporation of high-contrast patterns placed in the beam before and after scanning each slab position. These patterns are expected to enable stitching accuracy of less than 1 µm.

Additionally, we observed sample deformation at the borders due to radiation damage with the current pink X-ray beam centred at 25 keV. The monochromatic beam would be a more suitable choice. The upcoming APS Upgrade will provide the opportunity to work with higher energies (40–50 keV) at the bending-magnet beamline 2-BM. This advancement should enable the imaging of thicker slabs with reduced radiation damage.

As shown in Appendix *A*[App appa], suppressing laminography artefacts caused by insufficient Fourier spectrum coverage for large laminography angles (≥30°) can be achieved using iterative reconstruction with total variation regularization. Applying the iterative scheme to large data volumes may take a lot of time and resources. For instance, reconstructing a 2048^3^ volume with the ADMM approach, typically involving 64 outer and four inner iterations, requires computing the forward and adjoint laminography operators 256 times each. According to Table 1 ▸, computing one of these operators on 1 GPU takes about 20 s, resulting in a total reconstruction time exceeding 3 h. Fortunately, the data chunking scheme presented in this work can be adapted for multi-GPU computations across multiple nodes. This should lead to significant acceleration of experimental data reconstruction. We plan to optimize the code accordingly in our future work.

After successfully obtaining micrometre-resolution laminographic images of the mouse brain, our attention now shifts towards advancing techniques for nanometre resolution. This could involve utilizing the Projection X-ray Microscope instrument planned for construction with the APS Upgrade (Bean *et al.*, 2021 ▸). By cutting mouse brains into thinner slabs and handling significantly larger data volumes, these steps represent crucial progress towards achieving high-resolution imaging of the human brain.

## Figures and Tables

**Figure 14:**
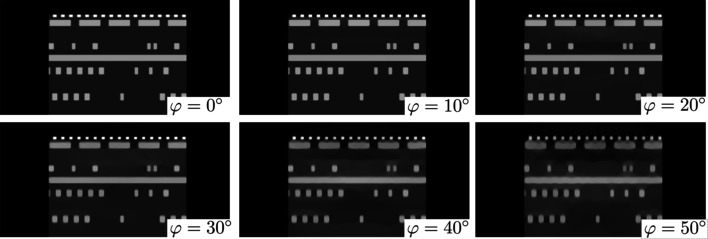
Results of reconstruction with total variation regularization for different laminography tilt angles. Data simulation parameters are the same as in Fig. 4 ▸.

**Table 1:** Time in seconds for processing *N* laminographic projections of size *N* × *N* to reconstruct *N* × *N* × *N* volumes ‘Data read’ – parallel read of 8-bit data from an SSD storage. ‘Pre-processing’ – dark-flat field correction, ring removal, Paganin filtering. ‘Reconstruction’ – reconstruction with the the *Fourierrec* (Fourier-based) method, and with the *Linerec* (direct discretization of the line integral) method. ‘Recon write’ – parallel write of 32-bit reconstructions to the SSD storage.

			Reconstruction				Total	
*N*	Data read (8-bit)	Pre-processing	*Fourierrec*	*Linerec*	Gain	Recon write (32-bit)	*Fourierrec*	*Linerec*
1024	1.3	1.9	3.0	9.3	3.1	4.2	10.4	16.7
1536	2.3	4.7	8.6	52.1	6.1	8.0	23.6	67.1
2048	4.6	7.8	21.1	222.1	10.5	18.1	51.6	252.6
3072	13.5	27.4	77.7	1002.2	12.9	52.3	170.9	1095.4
4096	31.1	59.4	164.0	4937.5	30.1	120.3	374.8	5148.3

## Appendix A Reconstruction with regularization

In this Appendix we demonstrate a scheme for laminographic reconstruction with total variation (TV) regularization. The scheme allows for suppressing laminography artefacts and improving the quality of results for the samples having a lot of features that are significantly different in amplitudes. We will consider the augmented Lagrangian formulation of the reconstruction problem and solve the problem by using ADMM (Boyd *et al.*, 2011 ▸) with splitting the whole problem by local sub-problems.

As in the previous section, let μ(*x*_1_, *x*_2_, *x*_3_) be a three-dimensional object and *d*(θ, *u*, *v*) its laminography data. Then the reconstruction problem with TV regularization reads as

with

where parameter α controls the trade-off between the data fidelity and regularization terms. The TV regularization promotes sparseness in reconstructions, resulting in noise suppression and incompleteness artefacts reduction (Chambolle & Pock, 2016 ▸). In particular, the ‘tail’ laminography artefacts are an example of data incompleteness in the frequency domain.

To solve the minimization problem (10) we first reformulate it as an equivalent constraint optimization problem with a new auxiliary variable ψ,

subject to ∇μ = ψ, and try to minimize the augmented Lagrangian written as follows,

where ρ > 0 is a penalty parameter and λ represents the dual variable. We use ADMM to split the minimization problem of the augmented Lagrangian into two local sub-problems with respect to μ and ψ. The sub-problems are then coordinated through variable λ to find a solution for the original problem. Specifically, the following steps are performed in each ADMM iteration *k*,

for zeros or some adequate initial guess at *k* = 0.

The minimization functional for the problem (14) can be written as follows,

where the terms not depending on μ are dropped. The problem is solved by considering the steepest ascent direction ∇_μ_*F*(*u*), given as

where the divergence operator div is the adjoint to −∇. With the steepest ascent direction, we can construct iterative schemes for solving (14) by using methods with different convergence rates. Here we employ the conjugate gradient (CG) method for its faster convergence rate at the expense of memory requirements. CG iterations are given as *u*_*m*+1_ = *u*_*m*_ + γ_*m*_η_*m*_, where γ_*m*_ is a step length computed by a line-search procedure (Nocedal & Wright, 2006 ▸) and η_*m*_ is the search direction that we compute by using the Dai–Yuan formula (Dai & Yuan, 1999 ▸),

where η_0_ = −∇_μ_*F*(μ_0_). On each ADMM iteration, we solve the tomography sub-problem approximately, by using only a few numbers of CG iterations since this strategy in practice greatly improves ADMM convergence rates – see review by Boyd *et al.* (2011 ▸).

The minimization problem with respect to ψ (15) has a closed form solution defined via the soft-thresholding operator (Donoho, 1995 ▸),

Fig. 14 ▸ demonstrates the results of solving the minimization problem (10) for different laminography tilt angles φ. Data simulation parameters for the integrated circuit dataset were the same as in Fig. 4 ▸. The regularization parameter α was chosen using the L-curve criterion (Agarwal, 2003 ▸).

Comparing reconstructed vertical slices presented in this figure with those presented in Fig. 4 ▸(*c*), one can see that the proposed TV regularization approach significantly helps in suppressing the ‘tail’ artefacts even for large laminography angles. It should be noted that the TV regularization for large values of the regularization parameter may also introduce cartoon-like artefacts (Chan & Shen, 2005 ▸). These artefacts are particularly seen in reconstruction for φ = 50° where we used a higher value of α than for other cases. It is generally advisable to search for a trade-off between the ‘tail’ and ‘cartoon’ artefacts with varying values of α.
